# Feasibility of balloon-based endobiliary radiofrequency ablation under cholangioscopy guidance in a swine model

**DOI:** 10.1038/s41598-021-93643-5

**Published:** 2021-07-09

**Authors:** Tadahisa Inoue, Hiromu Kutsumi, Mayu Ibusuki, Masashi Yoneda

**Affiliations:** 1 Department of Gastroenterology, Aichi Medical University, 1-1 Yazakokarimata, Nagakute, Aichi 480-1195 Japan; 2 Center for Clinical Research and Advance Medicine, Shiga University of Medical Science, Seta Tsukinowa-Cho, Otsu, Shiga 520-2192 Japan

**Keywords:** Gastroenterology, Medical research, Oncology

## Abstract

Although endobiliary radiofrequency ablation (RFA) has demonstrated considerable potential for the treatment of biliary strictures, conventional catheter RFA has several limitations. This study aimed to evaluate the feasibility of a novel cholangioscopy (CS)-guided balloon-based RFA procedure in vivo using a swine model. CS-guided balloon-RFA was performed under endoscopic retrograde cholangiography guidance at target temperatures of 60 ℃ or 70 ℃, which were maintained for 60 s. We evaluated the technical feasibility, adverse events, and histological effects associated with the procedure. Twelve sites were ablated in seven miniature pigs. The CS-guided balloon-RFA procedure was technically successful in all cases without any hindrance. Mucosal changes could be detected during RFA, and the ablation area was identified on CS. Necropsy was performed in four pigs on the same day as the procedure: the tissue samples showed coagulative necrosis, and the entire internal circumference of the bile duct was uniformly ablated. The mean lengths of the ablation area in the samples ablated at 60 °C and 70 °C were 20.64 and 22.18 mm, respectively, while the mean depths were 3.46 and 5.07 mm, respectively. The other three pigs were reared and euthanized and autopsied 35 days after the procedure. The site to be ablated had replaced the granulation tissue and fibrotic changes. No adverse events were observed in any case. CS-guided balloon-RFA appears to be a promising option for treating biliary strictures. This preliminary study could pave the way for the evaluation of this procedure in future human clinical trials.

## Introduction

Endobiliary radiofrequency ablation (RFA) is a new adjunctive procedure and promising treatment option for malignant biliary stricture^1,2^, which can prolong biliary stent patency and survival time^3–7^. However, the efficacy of endobiliary RFA remains controversial, and previous studies have reported conflicting results^8,9^, which could be attributed to a limitation of the conventional RFA catheter itself^10^: the ablation effect depends on each lesion’s characteristics, including the stricture length, and the area to be ablated cannot be minutely controlled in the conventional RFA method. Moreover, the safety of the procedure is similarly a concern because RFA cannot be performed under direct observation with ultrasound or endoscopic guidance.

To address these issues, we developed a balloon-based endobiliary RFA (balloon-RFA) system and observed that it could achieve a consistent ablation effect without excessive ablation and could be adapted to various lesions, as reported in an ex vivo study^11,12^. Another benefit of balloon-RFA is that it allows for the use of a narrower catheter diameter compared to conventional methods. This may allow for insertion through the working channel of a cholangioscope, enabling RFA under direct cholangioscopic guidance. This study aimed to evaluate the feasibility of this novel cholangioscopy (CS)-guided balloon-RFA procedure in vivo using a swine model.

## Methods

### CS-guided balloon-RFA system

**Figure 1:**
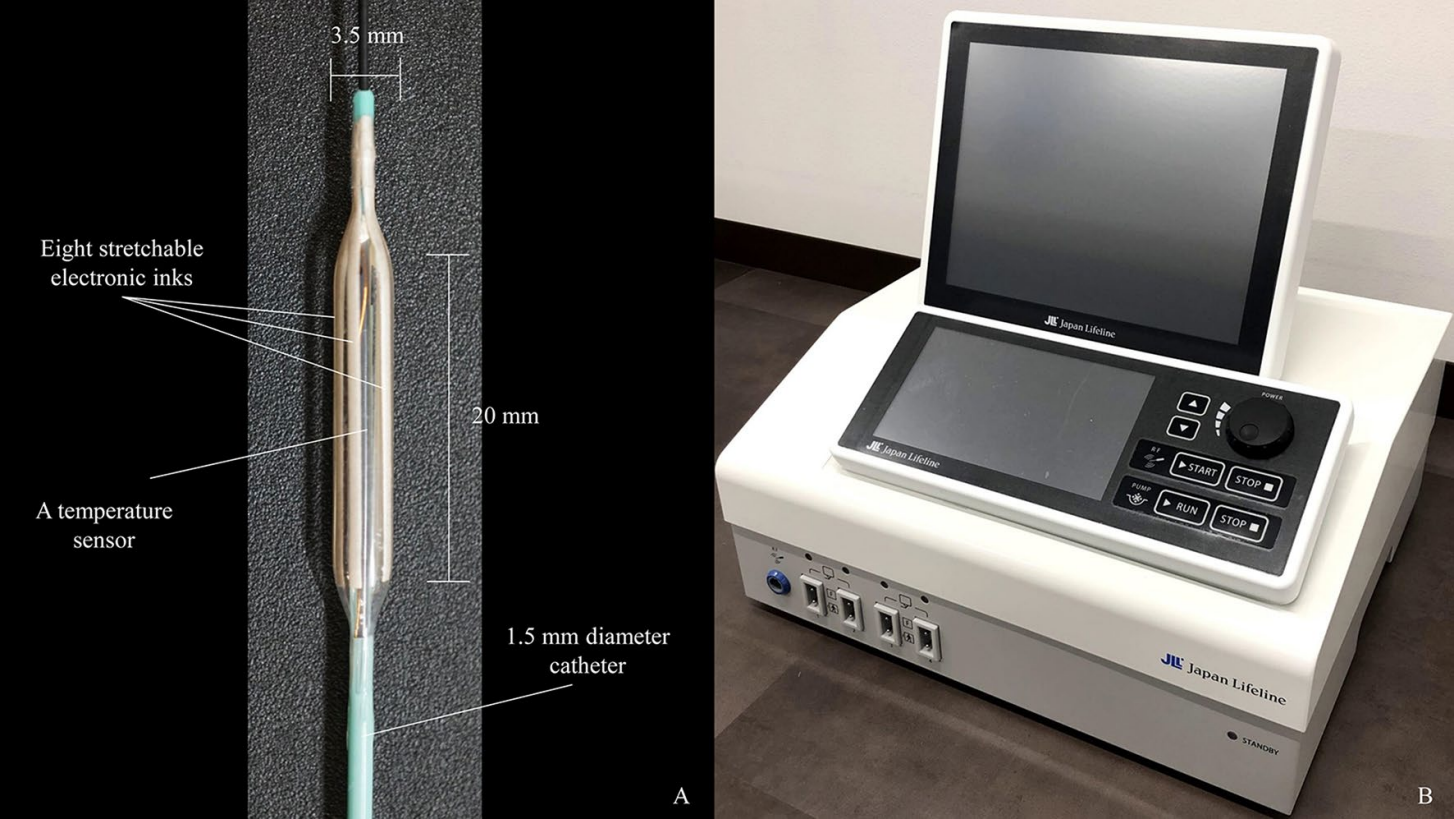
(**A**) Prototype balloon-based radiofrequency ablation catheter, which is ultrathin (1.5-mm diameter) to enable insertion through the working channel of a cholangioscope. It has a 3.5- or 6-mm-diameter balloon at the tip, eight stretchable electronic inks printed along the balloon’s length, and a temperature sensor at the surface of the balloon. (**B**) The generator can monitor temperature, power status, and impedance during the procedure in real-time.

This study utilized a prototype CS-guided balloon-RFA system (Japan Lifeline Co., Ltd., Tokyo, Japan) (Fig. 1). Two types of catheters were used: each catheter is equipped with a 3.5- or 6-mm-diameter balloon at the tip. The balloon length measured 20 mm in both types of catheters, and eight stretchable electronic inks are printed along the balloon’s length. The inks’ materials contain metal powder and plastic paste, which has high conductivity and stretching performance, and its resultant thickness is 5-μm. The catheter was selected according to the diameter of the bile duct that was to be subjected to ablation. The surface of the balloon is also equipped with a temperature sensor, and real-time monitoring of the impedance is equally possible during the procedure. The sensor is located in the center of the long axis of the balloon because the maximum temperature is recorded at the center of the electrodes when the balloon contacts the tissue evenly.

### Experimental procedure

The Institutional Animal Care and Use Committee of Aichi Medical University approved this study. The study was carried out in compliance with all relevant guidelines. Additionally, the ARRIVE guidelines have been followed for the study.

Seven miniature pigs with a bodyweight of 30–35 kg were used for this in vivo experimental study. They were premedicated with intramuscular injection of ketamine (10 mg/kg), xylazine (2 mg/kg), and atropine sulfate (0.5 mg). Subsequently, general anesthesia was administered and maintained using isoflurane (1–3%).

**Figure 2:**
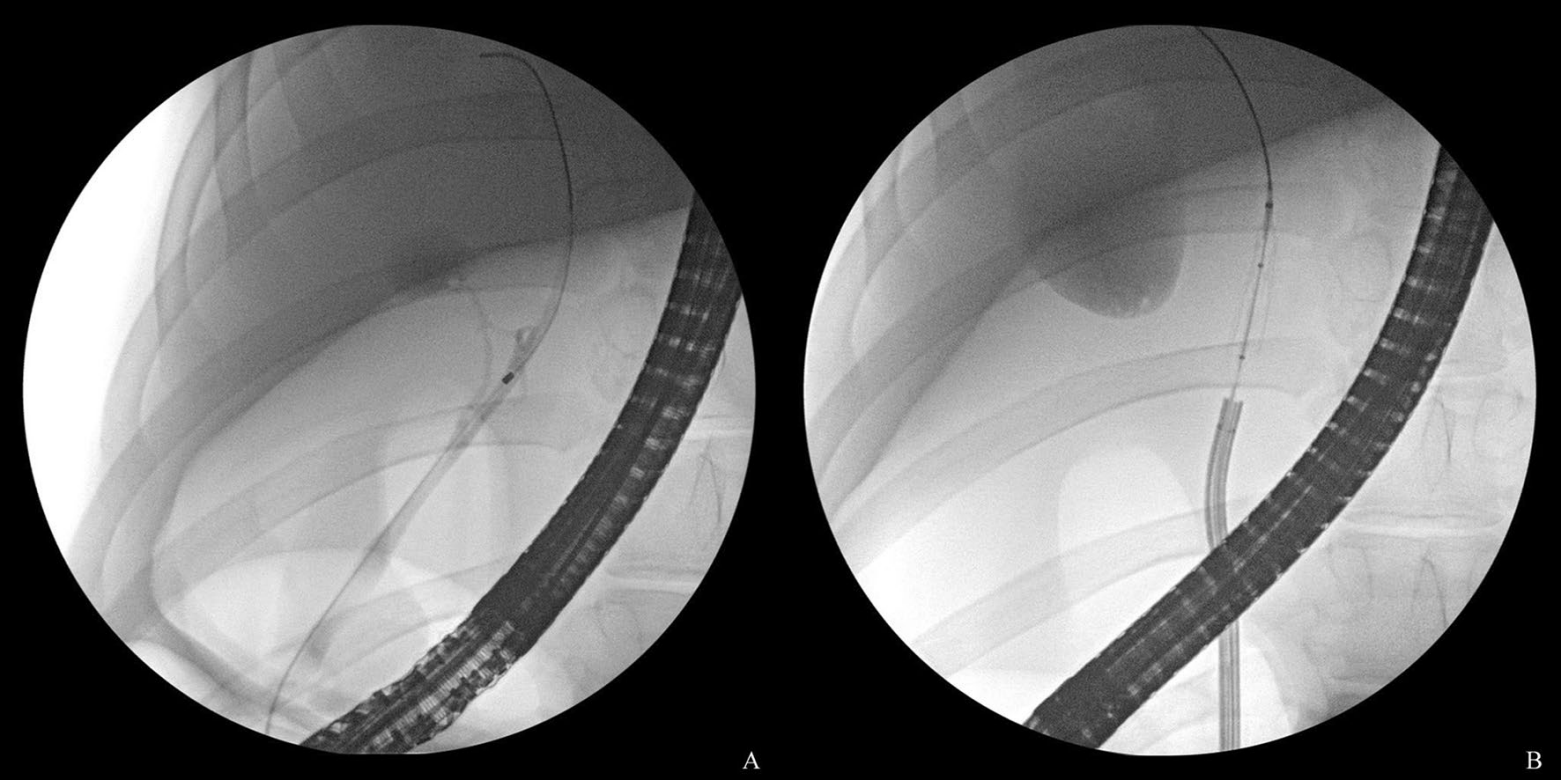
After obtaining the cholangiogram and achieving guidewire placement in the bile duct (**A**), a cholangioscope is inserted, and radiofrequency ablation with a balloon-based catheter is subsequently performed under cholangioscopic and fluoroscopic guidance (**B**).

**Figure 3:**
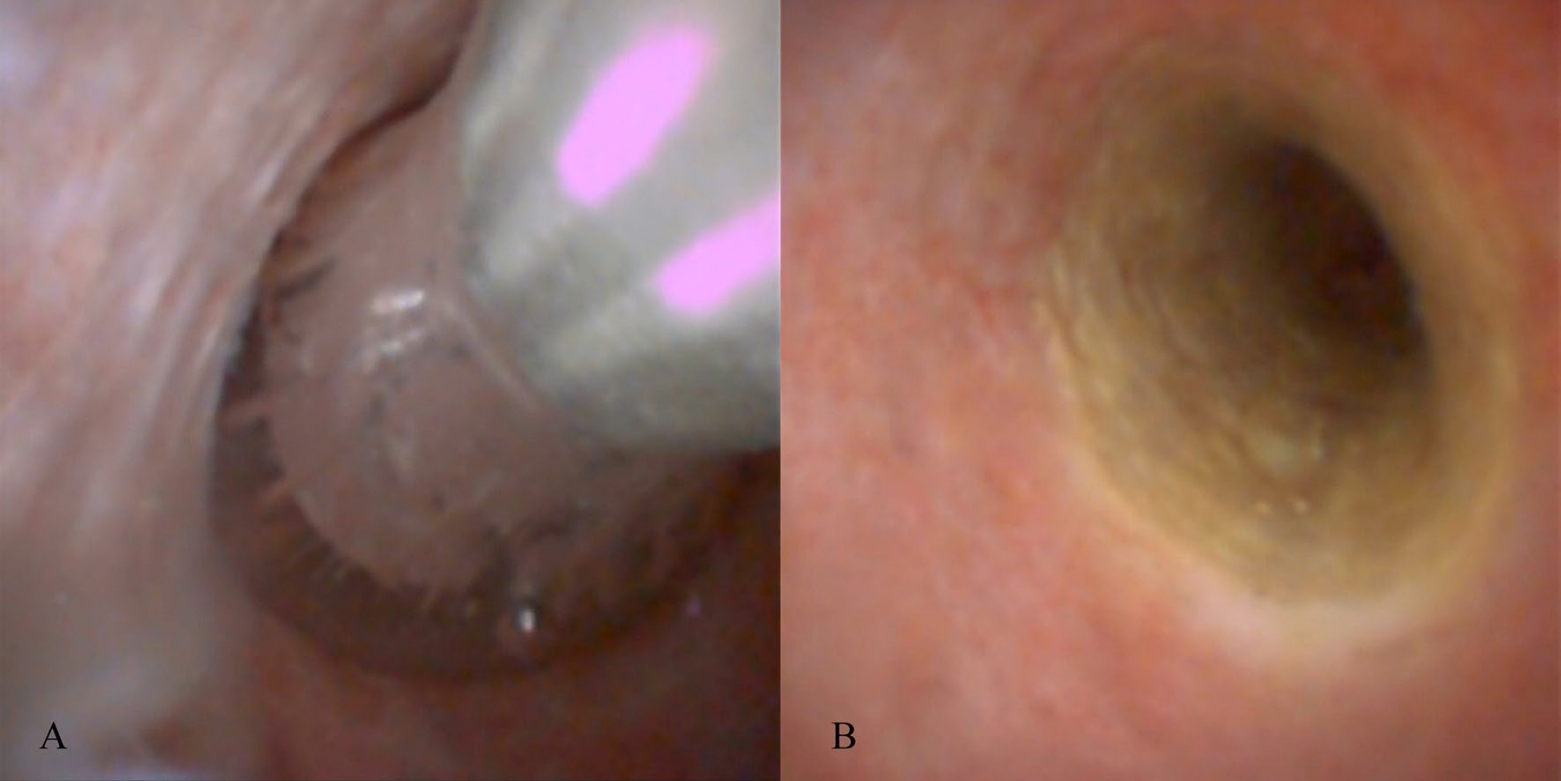
Cholangioscopy can confirm contact between the electrodes and bile duct wall (**A**) and clearly detect the ablation area after the removal of the catheter (**B**).

**Figure 4:**
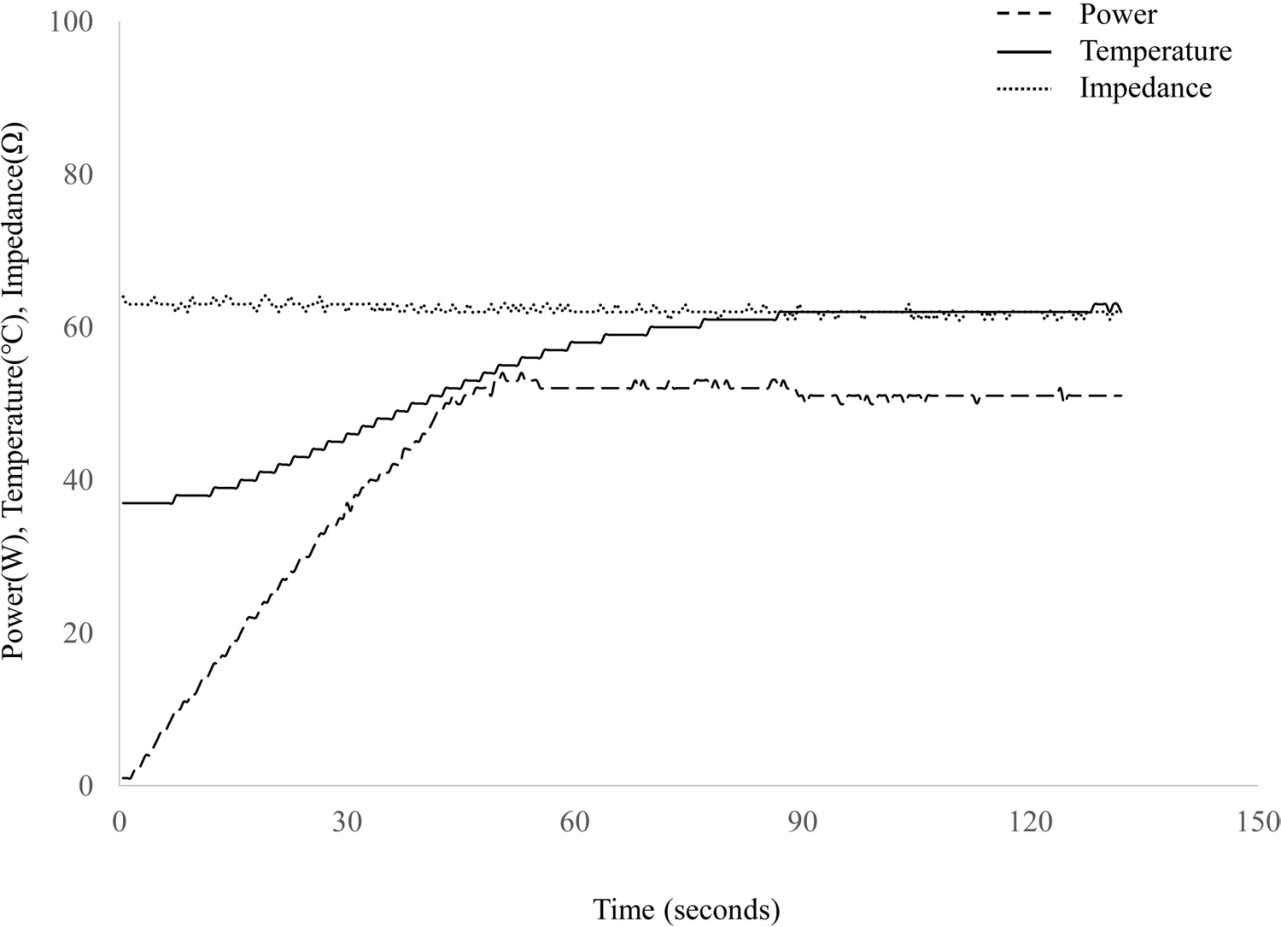
An example of the power, temperature, and impedance statuses during the ablation. The output power was increased linearly until the target temperature was reached and was adjusted continually to maintain the temperature.

The endoscopic procedure was as follows. Biliary cannulation was performed using a standard endoscopic retrograde cholangiopancreatography catheter with a 0.025-inch guidewire after a TJF-240 duodenoscope (Olympus Medical Systems, Tokyo, Japan) was advanced into the duodenal bulb. The cholangioscope was subsequently advanced over the guidewire, and the balloon-RFA catheter was inserted into its working channel. The balloon was inflated with water and placed in contact between the electrodes and bile duct wall under CS guidance. Ablations were subsequently performed at two different target temperatures (60 °C and 70 °C), which were maintained for 60 s while monitoring the procedure with the CS and fluoroscopy (Figs. 2, 3). The output power was increased linearly till the target temperature was reached and adjusted continuously to maintain the temperature while monitoring the impedance (Fig. 4). Based on the bile duct findings, including the length obtained on the cholangiogram, one to three ablations were performed for each pig. After ablation, the balloon was deflated and removed, and sufficient ablation of the bile duct wall and the presence or absence of adverse events, including bleeding and perforation, were confirmed. The view of the procedure through the CS is shown in Video 1.

### Histological analysis

After the procedure, the pigs were euthanized on the same day or 35 days later, and the bile duct and surrounding organs were removed en bloc. The bile duct was dissected longitudinally to expose the mucosal surface, and high-quality photographs were acquired to evaluate the ablation area. The resected ablated region and the adjacent tissue were fixed in formalin, embedded in paraffin, and sectioned for pathological analysis, which entailed staining with hematoxylin and eosin.

### Outcomes

The outcomes included technical success, adverse events, and histological effect associated with CS-guided balloon-RFA. Technical success was defined as the technical success of the procedure from cholangioscope insertion to the completion of the ablation. The incidence of adverse events was confirmed using cholangiography, CS, and gross and microscopic findings. The histological effects, including the length and depth of the ablation area, were evaluated both macroscopically and microscopically.

## Results

The experimental results are presented in Table 1. Twelve sites were ablated in seven pigs. All applications of the CS-guided balloon-RFA procedure were technically successful without any hindrance. The target temperature was not reached in two cases, where CS showed thick bile ducts and poor contact of the electrodes with the bile duct wall. Except for these two cases, mucosal changes during RFA could be detected on CS in all instances, and the ablation area was similarly identified immediately after ablation and the removal of the balloon-RFA catheter. Ablation led to distinct white-yellowish segmental changes in the bile duct wall. No procedure-related adverse events, including perforation and bleeding, were observed on CS and cholangiography performed after the ablation.

**Table 1:** Findings of cholangioscopy-guided balloon-based endobiliary radiofrequency ablation in an animal model.

Animal no.	Specimen no.	Sacrificed (day)	Bile duct site ablated	Balloon size	Target temperature	Ablation time^†^	Impedance	Technical success	Confirmation of mucosal changes during the ablation on CS	Accuracy of the ablation area by CS	Complete internal circumference ablation	Length of ablation area (mm)	Depth of ablation area (mm)	Adverse events
1	1–1	1	Superior	6.0 mm × 20 mm	60 ℃	60 s	63 Ω	Yes	Yes	Yes	Yes	20.05	3.76	None
	1–2	1	Inferior	6.0 mm × 20 mm	60 ℃	60 s	63 Ω	Yes	Yes	Yes	Yes	21.05	3.81	None
2	2–1	1	Superior	3.5 mm × 20 mm	(< 60 ℃)^‡^	–^‡^	92 Ω	Yes	No	Yes	No	Not obvious	0.21	None
	2–2	1	Inferior	6.0 mm × 20 mm	(< 60 ℃)^‡^	–^‡^	96 Ω	Yes	No	Yes	No	Not obvious	0.84	None
3	3–1	1	Superior	6.0 mm × 20 mm	70 ℃	60 s	65 Ω	Yes	Yes	Yes	Yes	21.25	5.29	None
	3–2	1	Inferior	6.0 mm × 20 mm	70 ℃	60 s	60 Ω	Yes	Yes	Yes	Yes	23.10	4.85	None
4	4–1	1	Superior	3.5 mm × 20 mm	60 ℃	60 s	68 Ω	Yes	Yes	Yes	Yes	20.07	3.10	None
	4–2	1	Middle	6.0 mm × 20 mm	60 ℃	60 s	64 Ω	Yes	Yes	Yes	Yes	20.01	4.39	None
	4–3	1	Inferior	6.0 mm × 20 mm	60 ℃	60 s	56 Ω	Yes	Yes	Yes	Yes	22.04	2.23	None
5	5–1	35	Superior	6.0 mm × 20 mm	60 ℃	60 s	80 Ω	Yes	Yes	-	Yes	19.05^§^	1.85^§^	None
6	6–1	35	Middle	6.0 mm × 20 mm	60 ℃	60 s	78 Ω	Yes	Yes	-	Yes	20.05^§^	3.02^§^	None
7	7–1	35	Superior	6.0 mm × 20 mm	60 ℃	60 s	67 Ω	Yes	Yes	-	Yes	21.00^§^	3.88^§^	None

*CS* cholangioscopy.

^†^Time for which the target temperature was maintained.

^‡^Did not reach the target temperature due to the thickness of the bile ducts and poor contact of the electrodes with the bile duct wall.

^§^These are the areas of granulation tissue rather than the ablation (coagulative necrosis) area.

**Figure 5:**
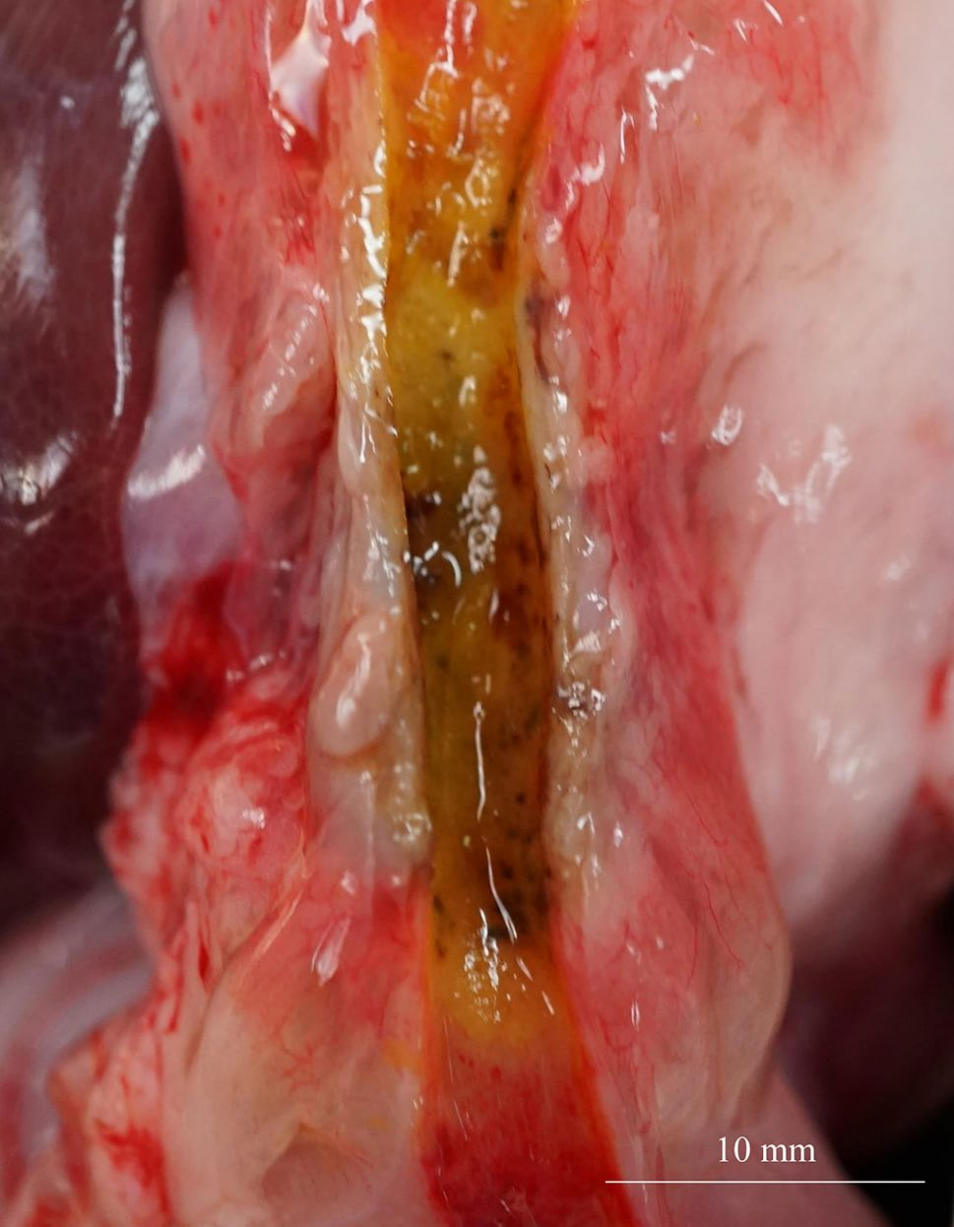
Macroscopic image of the bile duct after balloon-based radiofrequency ablation. The mucosa at the site of ablation appeared dark-brown compared to that surrounding the normal bile duct.

**Figure 6:**
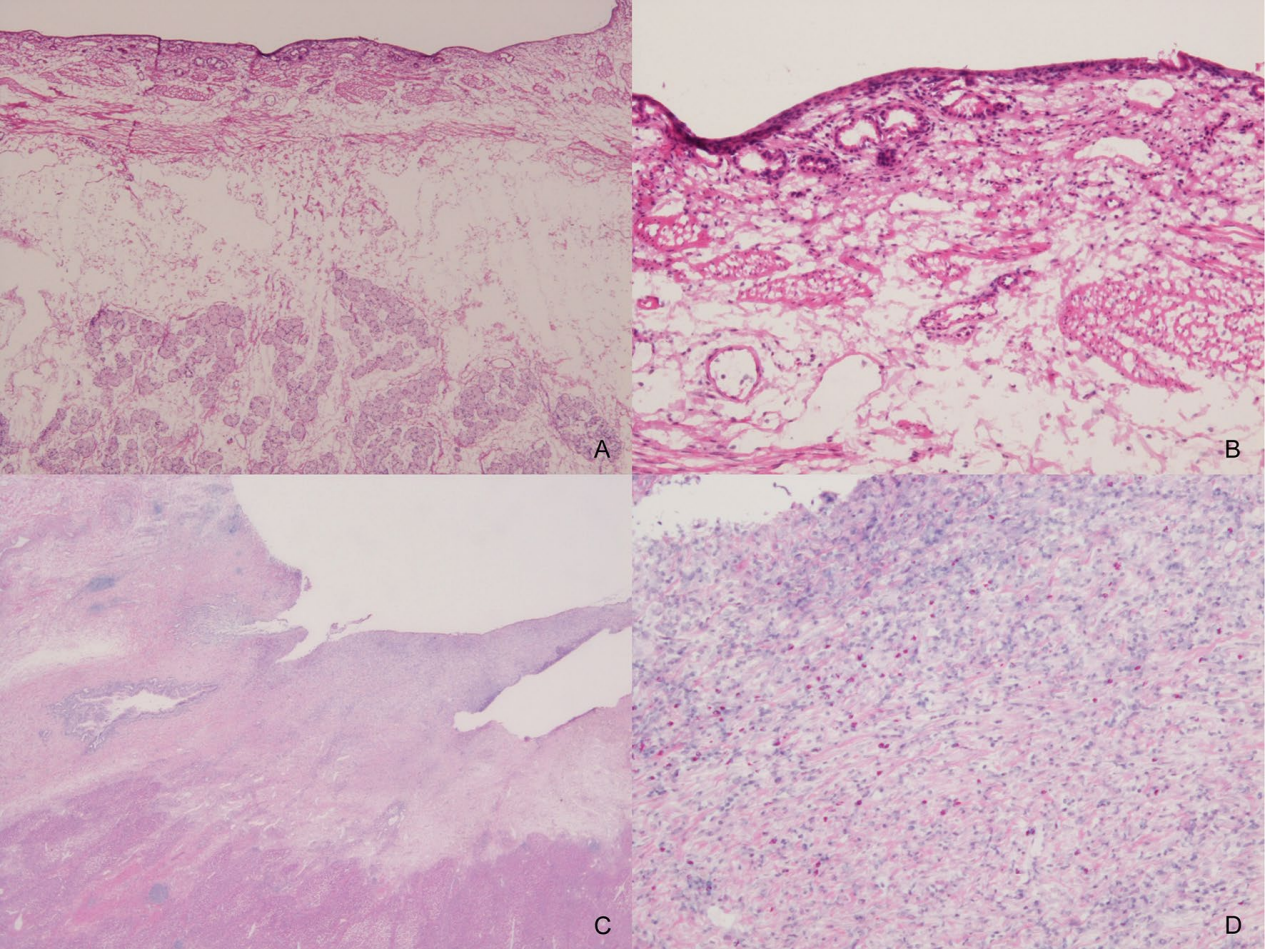
Photomicrographs of the histological sections of the bile duct after balloon-based radiofrequency ablation. The bile duct wall is thermally injured, and coagulative necrosis is observed at the bile duct wall and the surrounding tissue in specimens obtained on the same day as the procedure; tissues and cells affected by the ablation transform to a dry, dull eosinophilic area ((**A**) × 40 magnification; (**B**) × 200 magnification). The site to be ablated has replaced the granulation tissue and fibrotic changes in the specimens 35 days after the ablation; tissues affected by the ablation comprise proliferated myofibroblasts and collagen fibers ((**C**) × 20 magnification; (**D**) × 200 magnification).

Necropsy was performed for four pigs on the same day as the procedure to enable histological analysis. The mucosa at the site of ablation appeared dark-brown compared to that surrounding the normal bile duct on gross examination (Fig. 5). All histological samples showed epithelial sloughing and coagulative necrosis (Fig. 6A,B), and the entire internal circumference of the bile duct was evenly ablated, except for the two cases wherein the target temperature was not reached. The ablation areas were not different from those observed using the CS. The mean lengths of the ablation area of the samples ablated at 60 °C and 70 °C for 60 s were 20.64 and 22.18 mm, respectively. The mean depths of the samples ablated at 60 °C and 70 °C were 3.46 and 5.07 mm, respectively. Histological analysis did not reveal perforation in any case.

The other three pigs, which were reared and followed up after the procedure, were euthanized and autopsied 35 days after the procedure. The site to be ablated had replaced the granulation tissue and fibrotic changes (Fig. 6C,D). The mean length and depth of the granulation tissue area were 20.03 and 2.92 mm, respectively. No late adverse events, including perforation, bleeding, and microabscess, were observed in any of the specimens.

## Discussion

This study demonstrated that the novel CS-guided balloon-RFA procedure was technically feasible in an in vivo setting, achieving uniform ablation of the entire internal circumference of the bile duct without any adverse events. CS allowed for real-time observation of the ablation-induced mucosal changes and immediate identification of the ablation area after completing the procedure.

Although endobiliary RFA is a treatment option with a high potential for malignant biliary strictures, there is still no consensus on its utility and safety^1,2^. This may be related to the heterogeneity of previous studies, which included differences in the underlying disease, stricture location and status, and/or type of the stent used after RFA^3–9^. Moreover, conventional RFA catheters do not achieve effective ablation of strictures that are short, soft, non-tight, or rough due to insufficient contact with the tissue^10,11^. Similarly, they do not achieve even ablation because the points in contact with the electrodes will inevitably experience pronounced and deep burns^11^. In addition to performing further well-designed randomized controlled trials, improvements in the devices used are needed to evaluate and establish the utility of endobiliary RFA.

Theoretically, balloon-RFA can facilitate contact of the electrodes with the entire circumference of the duct, irrespective of the status of the stricture. This system can also monitor and maintain the temperature and monitor the impedance during the procedure, which makes it possible to minutely control the ablation. Therefore, the differences between the minimum and maximum ablation depths are significantly smaller with balloon-RFA than with conventional RFA, as previously shown in an ex vivo study (0.73-mm vs. 2.00-mm, P < 0.001)^11^. In this study, the ablation area consistently showed a cylindrical pattern around the catheter, with no adverse events observed. In the future, it is expected that the ablation depth may be set and controlled according to pre-RFA intraductal ultrasonography assessment. Although there were two cases of insufficient ablation because of poor contact of the electrodes with the bile duct wall associated with the thicker diameter of the pigs’ normal bile duct than the balloon diameter, there are probably no strictures wherein contact cannot be achieved by the 3.5- or 6-mm balloons.

Currently, endobiliary RFA can only be performed under fluoroscopy by endoscopic or percutaneous approach. Direct observation during the procedure is essential to obtain more reliable effects and maintain safety. In this study, the balloon-RFA catheter could be inserted through the working channel of the cholangioscope, enabling the observation of the ablation of the bile duct wall and identification of the ablation area in real-time. Therefore, CS-guided RFA could be useful in judging whether the electrodes are in appropriate contact with the tissue and determining the adequacy of ablation during the procedure, thus, allowing the endoscopist to judge whether to discontinue the procedure or extend the ablation time. Furthermore, the need for additional ablation and location of the area to be ablated could be determined by the CS findings immediately after the procedure.

The results of this study should be considered in the context of its limitations, which include the in vivo design using a normal swine model and a small number of swine. Animal models of biliary strictures do not exist, limiting the assessment of the effect of balloon-RFA; there may be a difference in resistance between health and cancerous tissues. Additionally, although the histological investigation was performed 35 days after the procedure, longer-term outcomes are unknown. Moreover, the efficacy and safety in human tissue, wherein the setting may be different to achieve a similar ablative depth, are still uncertain. Therefore, it is necessary that its utility and safety be confirmed in further analyses to establish this innovative procedure.

Despite these limitations, this is the first study to report this novel CS-guided balloon-RFA procedure, which demonstrated technical feasibility with safety and significant potential for the treatment of biliary strictures. This preliminary study could pave the way for further evaluation of this procedure in human clinical trials.

## Supplementary Information

Supplementary Legend.

Supplementary Video 1.
