# Supplementary material for: Feasibility of balloon-based endobiliary radiofrequency ablation under cholangioscopy guidance in a swine model
Source: Sci Rep. 2021 Jul 9;11:14254. doi: 10.1038/s41598-021-93643-5 (PMC8270987; doi:10.1038/s41598-021-93643-5)
Supplement: Supplementary file 1 — Supplementary Legend. [file 41598_2021_93643_MOESM1_ESM.docx]

**Supplementary Video 1**

Cholangioscopic view of the cholangioscopy-guided balloon-based endobiliary radiofrequency ablation procedure in a swine model
